# Tetrahedral framework nucleic acids promote diabetic wound healing via the Wnt signalling pathway

**DOI:** 10.1111/cpr.13316

**Published:** 2022-07-22

**Authors:** Zejing Wang, Hao Lu, Tao Tang, Lei Liu, Bohan Pan, Jiqiu Chen, Dasheng Cheng, Xiaoxiao Cai, Yu Sun, Feng Zhu, Shihui Zhu

**Affiliations:** ^1^ Burn Institute of PLA, Department of Burn Surgery the First Affiliated Hospital of Naval Medical University, Research Unit of Key Techniques for Treatment of Burns and Combined Burns and Trauma Injury, Chinese Academy of Medical Sciences Shanghai China; ^2^ State Key Laboratory of Oral Diseases National Clinical Research Center for Oral Diseases, West China Hospital of Stomatology, Sichuan University Chengdu China

## Abstract

**Objectives:**

To determine the therapeutic effect of tetrahedral framework nucleic acids (tFNAs) on diabetic wound healing and the underlying mechanism.

**Materials and Methods:**

The tFNAs were characterized by polyacrylamide gel electrophoresis (PAGE), atomic force microscopy (AFM), transmission electron microscopy (TEM), dynamic light scattering (DLS) and zeta potential assays. Cell Counting Kit‐8 (CCK‐8) and migration assays were performed to evaluate the effects of tFNAs on cellular proliferation and migration. Quantitative polymerase chain reaction (Q‐PCR) and enzyme‐linked immunosorbent assay (ELISA) were used to detect the effect of tFNAs on growth factors. The function and role of tFNAs in diabetic wound healing were investigated using diabetic wound models, histological analyses and western blotting.

**Results:**

Cellular proliferation and migration were enhanced after treatment with tFNAs in a high‐glucose environment. The expression of growth factors was also facilitated by tFNAs in vitro. During in vivo experiments, tFNAs accelerated the healing process in diabetic wounds and promoted the regeneration of the epidermis, capillaries and collagen. Moreover, tFNAs increased the secretion of growth factors and activated the Wnt pathway in diabetic wounds.

**Conclusions:**

This study indicates that tFNAs can accelerate diabetic wound healing and have potential for the treatment of diabetic wounds.

## Graphical abstract

Tetrahedral framework nucleic acids (tFNAs) can facilitate the proliferation and migration of HaCaTs, HDFs and HUVECs in a high‐glucose environment. tFNAs can accelerate diabetic wound healing by promoting epithelialization, vascularization, collagen synthesis and the secretion of growth factors via the Wnt pathway.

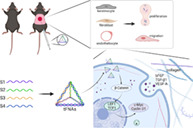

## 1 INTRODUCTION

Diabetes mellitus (DM) is one of the most serious global health problems owing to its prevalence and multiple complications.^1^, ^2^ Diabetic wounds are common and add to the complexity of DM through healing delays, multiple infections and diabetes‐related amputations.^3^, ^4^, ^5^ Because of the long cycle and high cost of wound treatment, there is an urgent need to explore effective and economical methods for the treatment of diabetic wounds.

The normal wound‐healing process in the skin involves a series of complex phases, including haemostasis, inflammation, proliferation and remodelling.^6^, ^7^, ^8^, ^9^ It requires the accurate interplay of multiple cells and mediators from the start. In DM, persistently high blood glucose levels affect the normal function of keratinocytes, fibroblasts and vascular endothelial cells.^3^, ^9^ Subsequently, the decreased production of growth factors, impaired angiogenesis, attenuated cellular proliferation and migration, continuous inflammatory response and abnormal collagen formation all contribute to chronic non‐healing wounds.^3^, ^10^, ^11^, ^12^ In addition, due to delayed wound healing, the local immune capacity is weakened and leaves wounds susceptible to infection, which eventually leads to tissue necrosis and amputation.^13^, ^14^ At present, the main treatments for diabetic wounds are blood glucose control, wound dressings and surgical debridement.^5^ Innovative strategies such as growth factor therapy, stem cell transplantation, genetic engineering and synthetic biomaterials are also being explored.^15^, ^16^ However, there is still a lack of convenient, rapid, economic and effective methods to treat diabetic wounds.

Since Nadrian Seeman designed the first DNA nanostructure in 1982, DNA nanotechnology has been rapidly developed and widely used.^17^ From two to three dimensions, DNA nanomaterials have been used in the bioimaging, biosensing, drug delivery and biomedical fields owing to their inherent biocompatibility, promising editability and spatial structural controllability.^18^, ^19^ Tetrahedral framework nucleic acids (tFNAs) are self‐assembled nucleic acid materials with three‐dimensional structures that are formed by four different single‐stranded DNAs (ssDNAs) based on the Watson‐Click base complementary pairing principle.^20^, ^21^ In contrast to ssDNAs and other DNAs, tFNAs can enter cells via caveolin‐mediated endocytosis and escape lysosome digestion.^22^ tFNAs have good biocompatibility, high biosafety, editability, nuclease resistance, simple synthesis and high yields.^23^, ^24^ Currently, tFNAs play an effective role in biosensing, regenerative medicine, cancer therapy and other fields.^25^, ^26^, ^27^ Previous studies have confirmed that tFNAs not only promote cell proliferation and migration but also have anti‐inflammatory, antioxidant and anti‐apoptotic effects.^28^ Therefore, tFNAs can promote angiogenesis, accelerate wound healing and improve the healing quality.^29^, ^30^ However, there have been few reports regarding the effects of tFNAs on diabetic wounds.

In this study, we evaluated the therapeutic role of tFNAs in diabetic wounds, both in vitro and in vivo. Our results confirmed that tFNAs promoted cellular proliferation and migration in a high‐glucose environment. Moreover, tFNAs facilitated epithelialization, vascularization, collagen synthesis and secretion of growth factors in diabetic wounds by activating the Wnt pathway.

## 2 MATERIALS AND METHODS

### 2.1 Synthesis and characterization of tFNAs

Four different ssDNAs were equally dissolved in TM buffer as described previously (sequences are shown in Table 1).^29^, ^30^ The mixture was denatured at 95°C for 10 min, cooled to 4°C for at least 20 min and stored at 4°C. The molecular weights of the single strands and tFNAs were determined by polyacrylamide gel electrophoresis (PAGE). Atomic force microscopy (AFM) and transmission electron microscopy (TEM) were used to determine the morphological structure of the tFNAs. A dynamic light scattering (DLS) assay was used to determine the particle size distribution of the tFNAs. A zeta potential assay was performed to determine the electric potential distribution of the tFNAs.

**TABLE 1:** Base sequences of the ssDNAs used to construct the tFNAs

ssDNA	Direction	Base sequence
S1	5′ → 3′	ATTTATCACCCGCCATAGTAGACGTATCACCAGGCAGTTGAGACGAACATTCCTAAG TCTGAA
S2	5′ → 3′	ACATGCGAGGGTCCAATACCGACGATTACAGCTTGCTACACGATTCAGACTTAGGAA TGTTCG
S3	5′ → 3′	ACTACTATGGCGGGTGATAAAACGTGTAGCAAGCTGTAATCGACGGGAAGAGCATGC CCATCC
S4	5′ → 3′	ACGGTATTGGACCCTCGCATGACTCAACTGCCTGGTGATACGAGGATGGGCATGCTC TTCCCG

### 2.2 Cell culture and groups

Human immortalized keratinocytes (HaCaTs) and human dermal fibroblasts (HDFs) were cultured in Dulbecco's modified Eagle's medium (DMEM; Gibco) supplemented with 10% (v/v) foetal bovine serum (FBS) and 1% (v/v) penicillin–streptomycin. Human umbilical vein endothelial cells (HUVECs) were cultured in F12 medium (Gibco) mixed with 10% (v/v) FBS and 1% (v/v) penicillin–streptomycin. To mimic the diabetic environment in vitro, cells were cultured under high glucose (40 mM) conditions for 72 h, and the medium was changed every 24 h. In the follow‐up experiments, the cells were grouped into three categories, normal cultured group (NC), high glucose cultured group (HG) and HG + tFNAs group. All the cells were cultured in an incubator in a controlled environment at 37°C and 5% CO_2_.

### 2.3 Cellular uptake of tFNAs

To confirm the cellular uptake of tFNAs in a high‐glucose environment, Cy5‐loaded tFNAs or Cy5‐loaded ssDNAs were mixed with the culture medium at a concentration of 250 nmol/L and then incubated with the cells. After 10 h of treatment, the cells were washed with phosphate‐buffered saline (PBS) and fixed with 4% (w/v) paraformaldehyde for 30 min. The cytoskeleton and nucleus were stained with phalloidin and DAPI (Cell Signaling Technology), respectively. Images were captured using a fluorescence microscope.

### 2.4 Proliferation assay

The Cell Counting Kit‐8 (CCK‐8; Dojindo, Japan) was used to assess cell proliferation. HaCaTs (8000 cells per well), HDFs (1500 cells per well) and HUVECs (2000 cells per well) were seeded in 96‐well plates (100 μl each well). After the cells were cultured for 4 h, cells in the HG + tFNAs group were treated with tFNAs at a concentration of 250 nmol/L, whereas those in the NC and HG groups were treated with PBS. Cell proliferation was examined after 24 h and 48 h of treatment. Ten microliters CCK‐8 solution was added to the 100 μl medium from each well before incubation for 4 h. The samples were examined at a wavelength of 450 nm.

### 2.5 Migration assay

Scratch experiments were performed to investigate the migratory behaviour of cells. For this, cells were seeded in 6‐well plates until they reached 100% confluency. After washing with PBS, a 200 μl pipette tip was used to create a cross‐shaped scratch in each well. The cells were washed three times and cultured in a medium containing different concentrations of tFNAs (0 or 250 nmol/L). At different time intervals, the area of the scratches was measured, recorded and compared with the original scratches at 0 h using the Image‐Pro Plus 6.0.

### 2.6 Enzyme‐linked immunosorbent assay (ELISA)

The secretion of basic fibroblast growth factor (bFGF), transforming growth factor‐β1 (TGF‐β1) and vascular endothelial growth factor‐A (VEGF‐A) from the HDFs and tissues of diabetic mice was analyzed using ELISA kits (Elabscience, China). Cells were seeded in 6‐well plates, incubated overnight and treated with tFNAs (0 or 250 nmol/L). After treatment for 24 h, supernatants were collected and subjected to ELISA in accordance with the manufacturer's protocol for qualitative assessment. The animal experiments are described below.

### 2.7 Quantitative polymerase chain reaction (Q‐PCR)

The expression levels of bFGF, TGF‐β1 and VEGF‐A were examined using Q‐PCR. Total cell RNA was extracted using the TRIzol reagent (Thermo Fisher). The mRNA samples were reverse‐transcribed into cDNA using a cDNA synthesis kit (Prime‐Script RT reagent Kit, Takara, Japan). Human β‐actin endogenous reference gene primers were purchased from Qing Xi Biological Technology (Shanghai, China). The primer sequences are listed in Table 2. Target genes were amplified by Q‐PCR using an ABI7300 (Thermo Fisher).

**TABLE 2:** Primer sequences for qPCR

Gene	Direction	Base sequence
bFGF	Forward(5′ → 3′)	TGGCTTCTAAATGTGTTACGG
	Reverse(5′ → 3′)	CGAATAAAGCAAATGCGTG
TGF‐β1	Forward(5′ → 3′)	CAATTCCTGGCGATACCTCAG
	Reverse(5′ → 3′)	GCACAACTCCGGTGACATCAA
VEGF‐A	Forward(5′ → 3′)	CATCCAATCGAGACCCTGGTG
	Reverse(5′ → 3′)	TTGGTGAGGTTTGATCCGCATA

### 2.8 Animal models

Diabetic BKS db/db mice (BKS – Leprem2Cd/Gpt, male, 8 weeks old) were purchased from the Model Animal Research Center of Nanjing University (Nanjing, China). All procedures complied with the guidelines of the Animal Committee of Changhai Hospital. The mice were anesthetized with 2% isoflurane and randomly divided into a control group and a tFNAs group. Full‐thickness excision skin wounds with a diameter of 8 mm were created on the backs of the mice. After surgery, the areas surrounding the wounds were subcutaneously injected with tFNAs (100 μl, 250 nmol/L, dissolved in saline) once a day for the next 7 days. The control group was injected with an equal volume of saline. Wound areas were photographed and recorded on days 0, 3, 7, 14 and 21. Skin tissue samples were collected for further study after the mice were euthanized. The wound healing rate (%) was calculated as follows:

$$\text{wound healing rate}\;\left(\%\right)=\left(1-\frac{\text{wound area}\;\mathrm{on}\;\mathrm{day}\;x}{\text{wound area}\;\mathrm{on}\;\mathrm{day}\;0}\right)\times 100\%$$

### 2.9 Histological analysis

Skin tissue samples from both the control and tFNAs groups were collected on day 21 after surgery. Additionally, normal skin samples from the diabetic mice were collected for comparison. After fixation with 4% paraformaldehyde, the samples were dehydrated, paraffin‐embedded, sectioned and stained with haematoxylin and eosin (HE), picrosirius red and Masson's trichrome. The tissue sections were observed and imaged using a microscope. Images were collected and quantified using the Image‐Pro Plus 6.0.

### 2.10 Immunohistochemistry and immunofluorescence

Tissue sections were incubated overnight with anti‐CD31 (D8V9E), anti‐α‐SMA (D4K9N), anti‐LEF1 (C12A5) and anti‐TCF1 (C63D9) antibodies (Cell Signaling Technology). After washing with PBS, the sections were incubated with secondary antibodies for 1 h. The stained sections were then exposed to DAPI for 10 min to stain the nuclei. Finally, the sections were observed and imaged using a fluorescence microscope.

### 2.11 Western blotting

The expression of β‐catenin, c‐Myc and cyclin D1 was examined by western blotting. After trituration, protein extraction and degeneration, the skin tissue samples were separated by SDS‐PAGE and transferred onto PVDF membranes. Membranes were placed in a blocking solution and incubated with the corresponding primary antibodies (anti‐β‐catenin (D10A8), anti‐c‐Myc (D84C12) and anti‐cyclin D1 (92G2); Cell Signaling Technology) at 4°C overnight. The next day, the membranes were incubated with secondary antibodies (BA1056; Boster, China) for 1 h at room temperature. Finally, the membranes were incubated with the exposure liquid and the protein bands were detected using a Bio‐Rad detection system (Hercules).

### 2.12 Statistical analysis

All data are presented as the mean ± standard deviation (SD). One‐way analysis of variance (ANOVA) and the Independent‐samples *t*‐test were used to determine the significance using the GraphPad Prism software (version 7.0). *p < 0.05, **p < 0.01 and ***p < 0.001 were considered thresholds of statistical significance.

## 3 RESULTS

### 3.1 Characterization of tFNAs and cellular uptake

**FIGURE 1:**
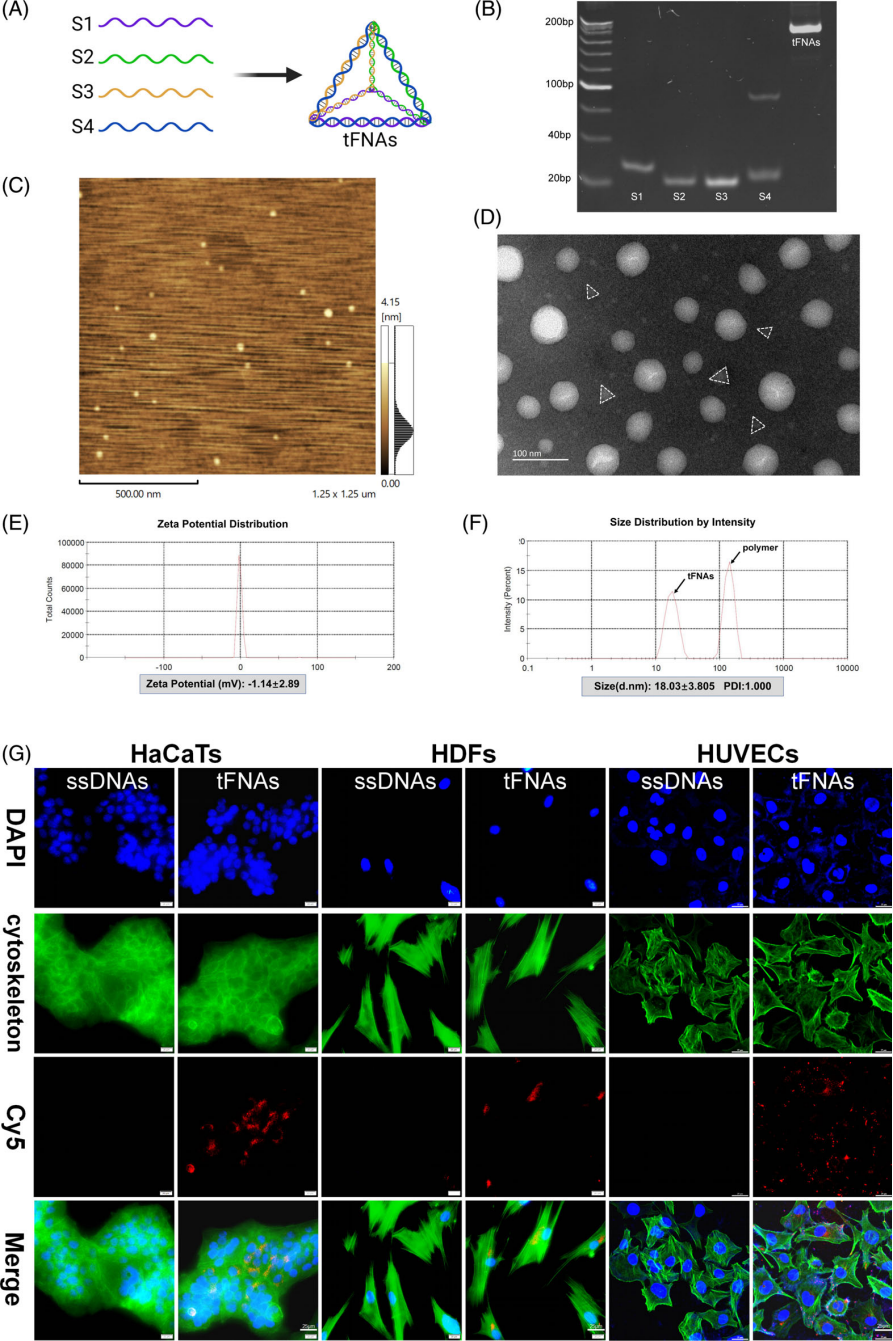
Preparation, characterization and cellular uptake of tFNAs. (A) Schematic illustration of the synthesis of tFNAs. (B) PAGE examination of the molecular weights. (C) AFM evaluation of the tFNAs. The scale bar is 500 nm. (D) TEM evaluation of the tFNAs. The scale bar is 100 nm. (E) Size distribution of the tFNAs. (F) Zeta potential distribution of the tFNAs. (G) Cellular uptake of tFNAs and ssDNAs (Cy5‐tFNAs and Cy5‐ssDNAs: red; nucleus: blue; cytoskeleton: green). Scale bars are 25 μm

The tFNAs were synthesized using four ssDNAs (S1, S2, S3 and S4) via specific base pairing (Figure 1A). PAGE was performed to examine the successful preparation of the tFNAs. The examination indicated that the molecular weights of ssDNAs were approximately 30 bp and those of tFNAs corresponded to approximately 200 bp, which was in accordance with the theoretical value of tFNAs (Figure 1B). AFM and TEM were used to evaluate the geometrical structure of the tFNAs; triangular structures were detected in the analyses (Figure 1C,D). In addition, polymers of the tFNAs were detected. The DLS peaks indicated that the average diameter of each tFNA was 18.03 ± 3.805 nm and the average diameter of polymeric tFNAs was 140.4 ± 24.41 nm (Figure 1E). The zeta potential assay showed that the nanoparticles were negatively charged and that the charge of the tFNAs was −1.14 ± 2.89 mV (Figure 1F).

To evaluate the ability of cells to uptake tFNAs and ssDNAs, HaCaTs, HDFs and HUVECs were treated with Cy5‐tFNAs or Cy5‐ssDNAs in a high‐glucose environment. The fluorescence intensity of the Cy5‐tFNAs was visibly stronger than that of Cy5‐ssDNAs (Figure 1G). The results revealed that tFNAs could easily be absorbed by these cells, whereas ssDNAs could not.

### 3.2 tFNAs promoted cell proliferation and migration in a high‐glucose environment in vitro

**FIGURE 2:**
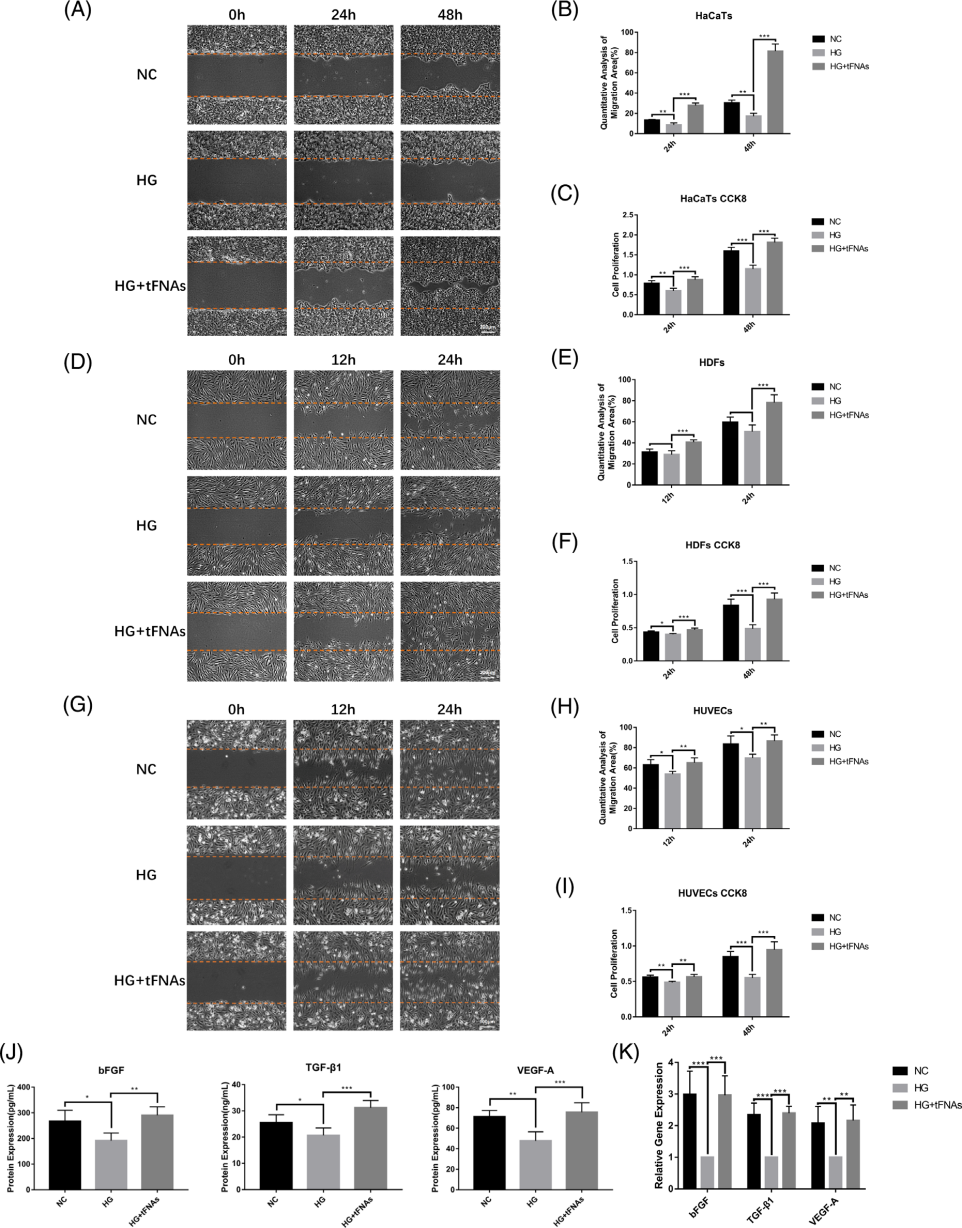
The effects of tFNAs on cellular proliferation, migration and growth factors. (A,B) In vitro wound‐healing assay of HaCaTs and semi‐quantification of the migration areas. The scale bar is 200 μm. (c) CCK‐8 results from HaCaTs. (D,E) In vitro wound‐healing assay of HDFs and semi‐quantification of the migration areas. The scale bar is 200 μm. (F) CCK‐8 results from HDFs. (G,H) In vitro wound‐healing assay of HUVECs and semi‐quantification of the migration areas. The scale bar is 100 μm. (I) CCK‐8 results from HUVECs. (J) ELISA results of bFGF, TGF‐β1 and VEGF‐A secretion at 24 h. (K) Transcript‐level expression of bFGF, TGF‐β1 and VEGF‐A by Q‐PCR at 24 h. Data are presented as the mean ± standard deviation (SD) (*n* = 5). Significance: *p < 0.05, **p < 0.01, ***p < 0.001

Keratinocytes, fibroblasts and vascular endothelial cells play an important role in wound healing.^31^, ^32^ To explore whether tFNAs influence the biological behaviour of the three cell lines, CCK‐8 and scratch experiments were performed to determine cell proliferation and migration in a high‐glucose environment.^33^, ^34^, ^35^ The results of the CCK‐8 assay showed that the proliferation of HaCaTs, HDFs and HUVECs was inhibited in a high‐glucose environment compared with that in normal culture. After treatment with tFNAs, the three cell types had greater proliferative ability than those in the HG group. The differences between the groups were more significant at 48 h than at 24 h after treatment (Figure 2C,F,I). In terms of migratory ability, the results indicated that the migratory rates of HaCaTs and HUVECs were attenuated in a high‐glucose environment (Figure 2B,H), whereas those of HDFs were not affected (Figure 2E). After treatment with tFNAs, the migratory abilities of the three cell types were enhanced in a high‐glucose environment (Figure 2A,D,G). The migration area of the HaCaTs in the HG + tFNAs group was approximately four times that of those in the HG group at 48 h (Figure 2B). These results confirmed that tFNAs promoted the proliferation and migration of HaCaTs, HDFs and HUVECs in a high‐glucose environment.

### 3.3 tFNAs facilitated the expression and secretion of growth factors in a high‐glucose environment in vitro

Wound healing involves the regulation of multiple growth factors, including the bFGF, TGF‐β1 and VEGF‐A.^31^, ^32^, ^36^ Therefore, ELISA and Q‐PCR were used to evaluate the expression (at the transcript level) and secretion of bFGF, TGF‐β1 and VEGF‐A under the influence of tFNAs in a high‐glucose environment. As shown in Figure 2J, the secretion of bFGF, TGF‐β1 and VEGF‐A in HDFs was reduced in cells of the HG group and recovered after treatment with tFNAs. Q‐PCR further confirmed that the transcript‐level expression of bFGF, TGF‐β1 and VEGF‐A was significantly decreased in a high‐glucose environment, and that tFNAs could reverse this trend (Figure 2K).

### 3.4 tFNAs accelerated diabetic cutaneous wound healing in vivo

To evaluate the therapeutic effect of tFNAs on diabetic wound healing in vivo, diabetic cutaneous wounds were created in BKS db/db mice and then randomly divided into control and tFNAs groups.

**FIGURE 3:**
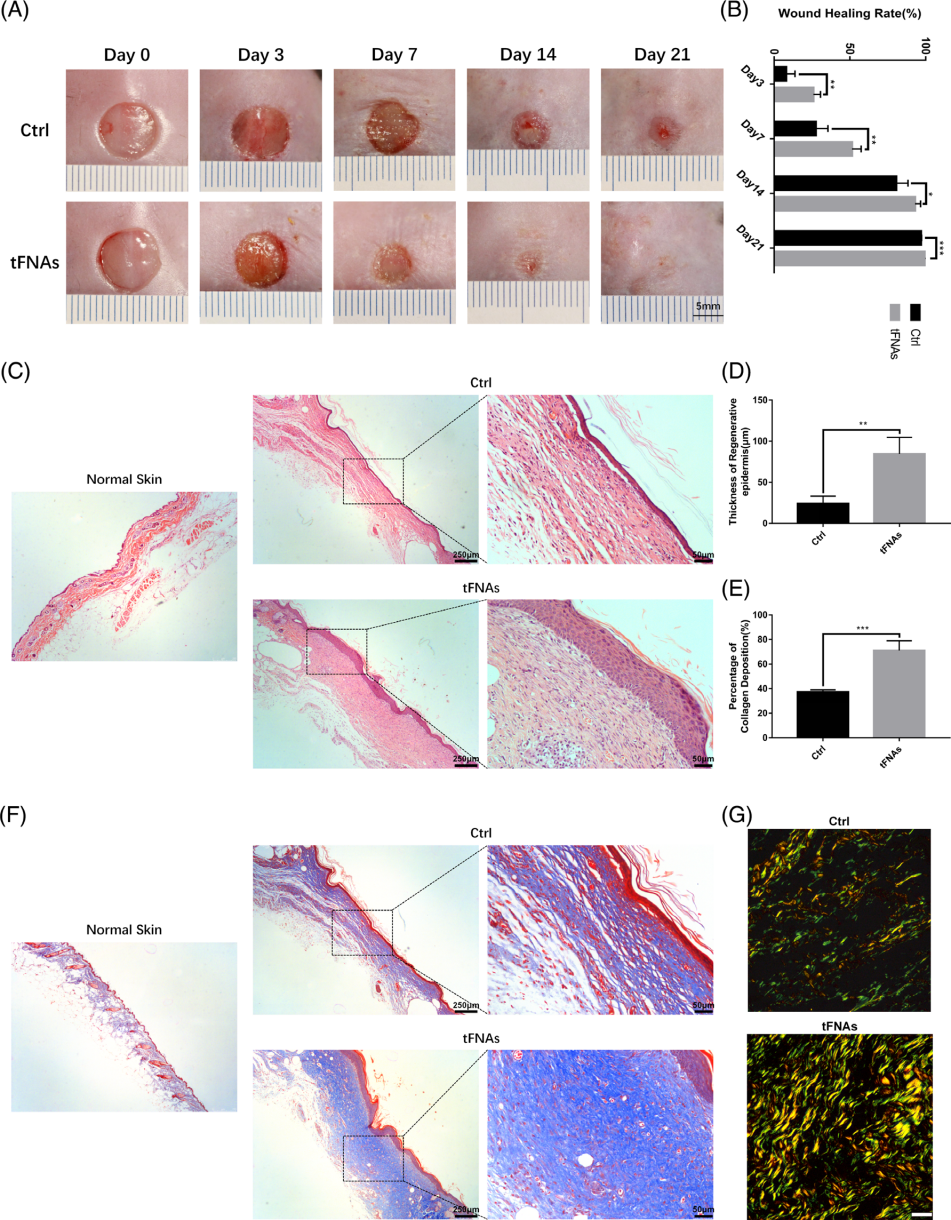
tFNAs facilitated diabetic wound healing, and increased epithelialization and collagen deposition in diabetic wounds. (A) Photographs of cutaneous wounds in diabetic mice treated with saline or 250 nM tFNAs at different time points. The scale bar is 5 mm. (B) Calculation and comparison of the wound healing area percentages between the control and tFNAs groups at different time points. (C) H&E staining of the control and tFNAs groups on day 21 after surgery. Scale bars are 250 or 50 μm. (D) Comparison of the average regenerative epidermal thickness between the control and tFNAs groups. (E) Comparison of the deposited collagen percentages between the control and tFNAs groups. (F) Masson staining of the control and tFNAs groups on day 21 after surgery. Scale bars are 250 or 50 μm. (G) Picrosirius red staining of the control and tFNAs groups on day 21 after surgery. The scale bar is 25 μm. Data are presented as the mean ± SD (*n* = 4). Significance: *p < 0.05, **p < 0.01, ***p < 0.001

The results in Figure 3A revealed that wound healing was accelerated in diabetic mice after treatment with tFNAs. The wound areas in the tFNAs group were significantly smaller than those in the control group on days 7 and 14 after surgery. By day 21, the wounds of the tFNAs group had been completely covered with the epidermal tissue, while the control group had a small number of residual wounds. Further statistical analysis (Figure 3B) showed that the wound‐healing rate in the tFNAs group was significantly higher than that in the control group on days 3, 7, 14 and 21 after surgery.

HE and Masson's staining showed that the regenerated skin tissues of the tFNAs group were more mature (Figure 3C,F), and that the epidermal thickness and deposited collagen percentages in the tFNAs group were significantly higher than those in the control group on day 21 after surgery (Figure 3D,E). Furthermore, the collagen fibres in the dermis of the tFNAs group were more compact and organized, whereas those in the control group were relatively loose (Figure 3F). Picrosirius red staining revealed that the number of yellow and green collagen fibres in the tFNAs group was higher than that in the control group (Figure 3G).

**FIGURE 4:**
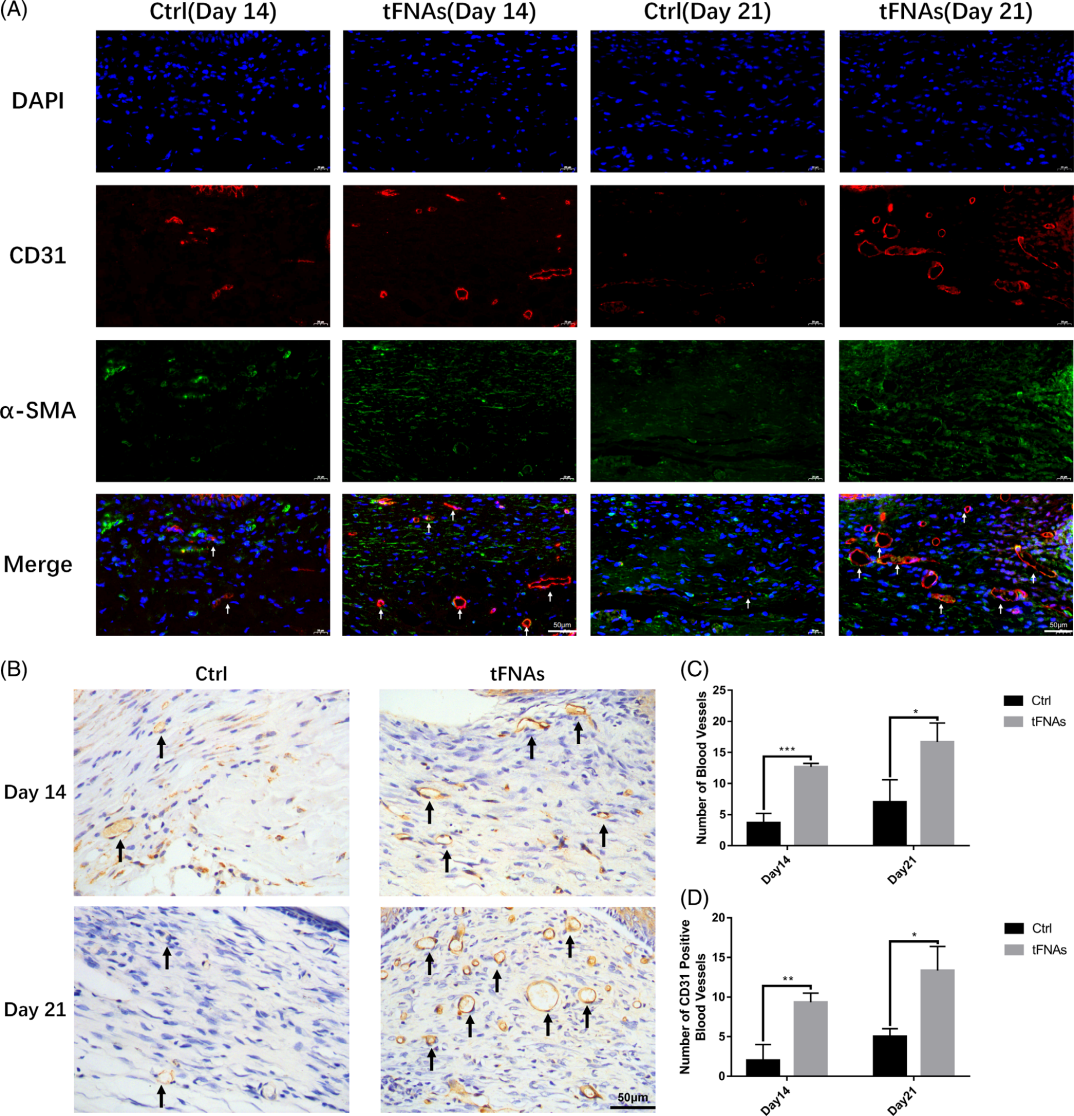
tFNAs increased vascularization in diabetic wounds. (A) Immunofluorescence staining of CD31 and α‐SMA expression of the control and tFNAs groups (white arrow: vessel; nucleus: blue; CD31: red; α‐SMA: green). Scale bars are 50 μm. (B) Immunohistochemical staining of CD31 expression of the control and tFNAs groups (black arrow: vessel). The scale bar is 50 μm. (C) Quantification analysis of the number of CD31‐ and α‐SMA‐positive blood vessels between the control and tFNAs groups. (D) Quantification analysis of the number of CD31‐positive blood vessels between the control and tFNAs groups. Data are presented as the mean ± SD (*n* = 3). Significance: *p < 0.05, **p < 0.01, ***p < 0.001

Angiogenesis is also a crucial factor affecting wound healing and provides sufficient nutrition to support the healing process.^37^ CD31 and α‐SMA are important blood vessel markers and can be used as indicators of neovascularization.^38^, ^39^, ^40^ Therefore, to explore whether tFNAs could facilitate neovascularization, CD31 immunohistochemistry and immunofluorescence of CD31 and α‐SMA were used to observe the blood vessels. Figure 4B,D revealed that there were more new blood vessels in the CD31‐positive diabetic wounds treated with tFNAs compared to those in the control group on days 14 and 21 after surgery. These results were further confirmed by immunofluorescence (Figure 4A,C).

**FIGURE 5:**
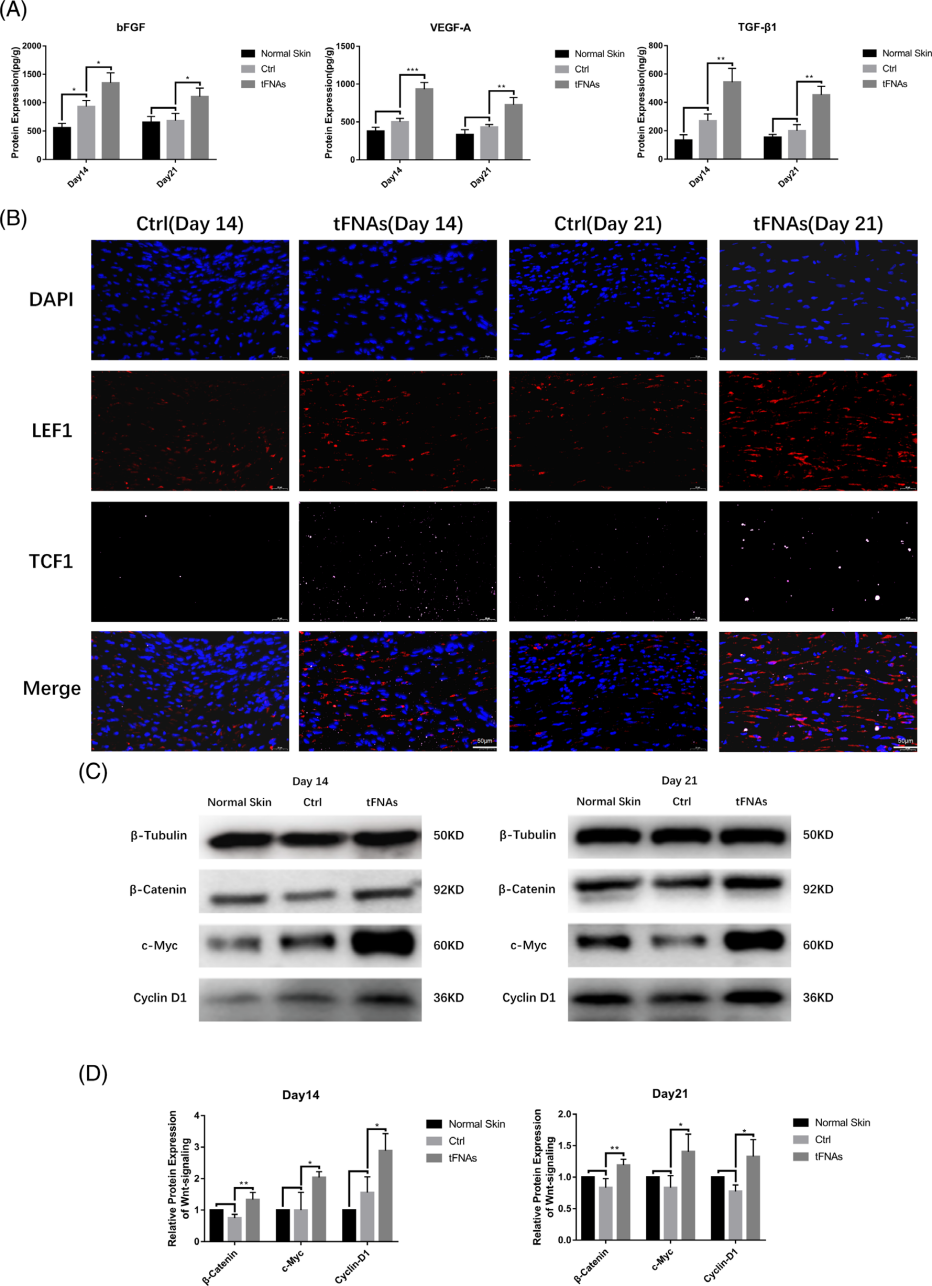
tFNAs regulated the secretion of growth factors and the Wnt signalling pathway in diabetic wounds. (A) ELISA results of bFGF, TGF‐β1 and VEGF‐A secretion in the control and tFNAs groups. (B) Immunofluorescence to check for the expression of LEF1 and TCF1 in the control and tFNAs groups (nucleus: blue; LEF1: red; TCF1: pink). Scale bars are 50 μm. (C) Western blot detection of β‐catenin, c‐Myc and cyclin D1 expression in the control and tFNAs groups. (D) Semi‐quantification of Wnt signalling pathway protein levels in (C). Data are presented as the mean ± SD (*n* = 3). Significance: *p < 0.05, **p < 0.01, ***p < 0.001

ELISA results of the tissue samples (Figure 5A) indicated that the secretion of bFGF in diabetic wounds was slightly increased compared to that in the normal skin on day 14 after surgery, whereas no difference was observed in the secretion of TGF‐β1 and VEGF‐A. On day 21, there were no differences in the levels of bFGF, TGF‐β1 and VEGF‐A between diabetic wounds and normal skin. However, after treatment with tFNAs, the secretion of bFGF, TGF‐β1 and VEGF‐A in diabetic wounds increased significantly.

In summary, animal experiments revealed that tFNAs could facilitate the healing of diabetic cutaneous wounds by promoting epithelialization, vascularization, collagen synthesis and secretion of growth factors.

### 3.5 tFNAs activated the Wnt pathway in diabetic wounds

The Wnt pathway plays a prominent role in diabetic wound healing^3^ and can promote epithelialization and improve fibroblast function and angiogenesis.^41^ β‐catenin is the key effector molecule of the Wnt pathway. LEF1 and TCF1 are crucial transcription factors, whereas c‐Myc and cyclin D1 are downstream molecules that regulate the cell state and promote tissue repair.^42^, ^43^ Considering the close relationship between the results of previous experiments and the function of the Wnt pathway, we assumed that the Wnt pathway is an essential component of the mechanism by which tFNAs promote diabetic wound healing. Western blotting indicated that the levels of β‐catenin, c‐Myc and cyclin D1 in diabetic wounds were not markedly changed compared to those in the normal skin on days 14 and 21 after surgery. Nevertheless, tFNAs significantly promoted the expression of β‐catenin, c‐Myc and cyclin D1 in diabetic wounds and activated the Wnt pathway (Figure 5C,D). Immunofluorescence revealed that the expression of LEF1 and TCF1 increased in the diabetic wounds treated with tFNAs compared to that in the control group on days 14 and 21 after surgery (Figure 5B).

## 4 DISCUSSION

As the body's first barrier against external injury, the skin can maintain internal homeostasis^44^; therefore, it is essential to repair the damaged skin in time. Many factors can affect wound‐healing, including age, nutritional status, infection, chemoradiotherapy and DM.^45^ An increasing number of patients suffering from diabetic wounds has resulted in a rise in associated health problems and medical burdens. Although some therapies, such as negative pressure therapy, stem cell therapy and various synthetic biological dressings, have exhibited beneficial effects,^15^, ^16^, ^46^, ^47^ they are expensive and require more complex operations.

DNA nanotechnology has rapidly developed in recent years and has been widely used in the biomedical field.^48^, ^49^ As a novel DNA nanomaterial, tFNAs can improve cell function and promote tissue regeneration.^18^, ^28^ Previous studies have confirmed that tFNAs can effectively enter cells and promote angiogenesis and wound healing.^29^, ^30^ Therefore, considering the obstructive factors of diabetic wounds and the functions of tFNAs, we explored the effects of tFNAs on diabetic wound healing. In our research, tFNAs were successfully assembled by base pairing. The size, shape, molecular weight and electric charge of the tFNAs were consistent with the characteristics reported in previous studies. In addition, our study demonstrated that tFNAs could be readily absorbed by HaCaTs, HDFs and HUVECs in a high‐glucose environment.

Wound healing involves the cooperation of various cell types. The proliferation and migration of keratinocytes directly affect wound sealing. Fibroblasts can regulate the healing process by secreting a variety of extracellular matrix such as collagen and fibronectin. Adequate angiogenesis can ensure the support of oxygen and nutrition during wound healing.^29^, ^31^, ^32^ Therefore, the functional status of keratinocytes, fibroblasts and vascular endothelial cells is crucial for wound healing. In our study, a high‐glucose environment directly affected the proliferation and migration of HaCaTs and HUVECs, as well as the proliferation of HDFs. Under the action of tFNAs, the proliferation and migration of the three cell types were significantly enhanced in a high‐glucose environment.

Cytokines also directly influence wound healing, including growth factors, inflammatory factors and chemokines.^36^ bFGF can promote granulation tissue formation, epithelialization, angiogenesis and tissue remodelling.^37^, ^50^ As a member of the TGF‐β family, TGF‐β1 regulates the functions of fibroblasts and keratinocytes and promotes collagen synthesis and tissue repair during the early stages of wound healing.^31^, ^51^ VEGF‐A is a strong angiogenic factor that accelerates the proliferation and migration of vascular endothelial cells.^36^ Studies have shown that a decrease in local growth factors is one of the reasons for refractory diabetic wounds.^52^ In our study, the ability of HDFs to secrete growth factors was attenuated in a high‐glucose environment. Animal experiments further revealed that the secretion of bFGF, TGF‐β1 and VEGF‐A was insufficient after the occurrence of skin lesions in diabetic mice. After treatment with tFNAs, the expression and secretion of bFGF, TGF‐β1 and VEGF‐A improved in the diabetic wound environment in vitro and in vivo.

To confirm the therapeutic effect of tFNAs on diabetic wound healing, diabetic BKS db/db mice were used in this study. The results showed that the wound‐healing rate of diabetic mice was significantly accelerated after treatment with tFNAs in the first 2 weeks after surgery. Epithelialization of diabetic wounds treated with tFNAs was completed on day 21 after surgery, while the wounds of the control group had not healed entirely. The formation of collagen plays a critical role in wound healing; however, this process is suppressed in diabetic wounds.^3^ In our study, the collagen content of diabetic wounds was significantly increased after treatment with tFNAs, and well‐organized collagen fibres were found in diabetic wounds treated with tFNAs. In addition, the regenerated epidermis was thicker and more solid under the effect of the tFNAs. Adequate blood vessels provide oxygen, nutrients, growth factors and cells for wound healing. As mentioned previously, neovascularization is attenuated and damaged in DM.^6^, ^12^ Immunohistochemistry and immunofluorescence revealed that more blood vessels were present in diabetic wounds treated with tFNAs compared to that in the control group. In summary, tFNAs could facilitate epithelialization, vascularization and collagen synthesis in diabetic wounds.

Previous studies have indicated that tFNAs can regulate multiple signalling pathways and produce different biological effects.^30^ The Wnt signalling pathway is a conserved signal transduction pathway that involves a variety of biological processes, including embryonic development, homeostasis and tissue regeneration.^3^, ^53^ It promotes cutaneous wound healing by regulating the functions of various cells and accelerating angiogenesis, epithelialization and dermal matrix formation.^41^, ^54^ Upregulation of the Wnt signalling pathway can facilitate diabetic wound healing.^3^, ^55^ Our results showed that the levels of β‐catenin, c‐Myc and cyclin D1 did not change after skin injury in diabetic mice, and that the Wnt pathway was not upregulated. After treatment with tFNAs, a pronounced increase in the expression of these proteins indicated that the Wnt pathway was activated in diabetic wounds. The levels of LEF1 and TCF1 were also increased in diabetic wounds treated with tFNAs. β‐catenin is the key effector molecule of the Wnt pathway that can bind to LEF1/TCF1 in the nucleus. These transcription factors regulate the expression of numerous downstream molecules, such as c‐Myc and cyclin D1; c‐Myc stimulates cells and increases proliferation, whereas cyclin D1 is an important protein in the cell cycle.^3^, ^42^, ^43^ Activation of the Wnt pathway can improve the function of various cells, including keratinocytes, fibroblasts and vascular endothelial cells. Subsequently, epithelialization, vascularization and collagen synthesis were enhanced in diabetic wounds. Moreover, with the promotion of cellular proliferation and function, additional growth factors are secreted to accelerate diabetic wound healing. This inference was consistent with our previous experimental results. Thus, the Wnt pathway may be an essential mechanism underlying the therapeutic effect of tFNAs in diabetic wound healing. However, the specific molecular interactions between tFNAs and intracellular molecules need to be verified in future studies.

**FIGURE 6:**
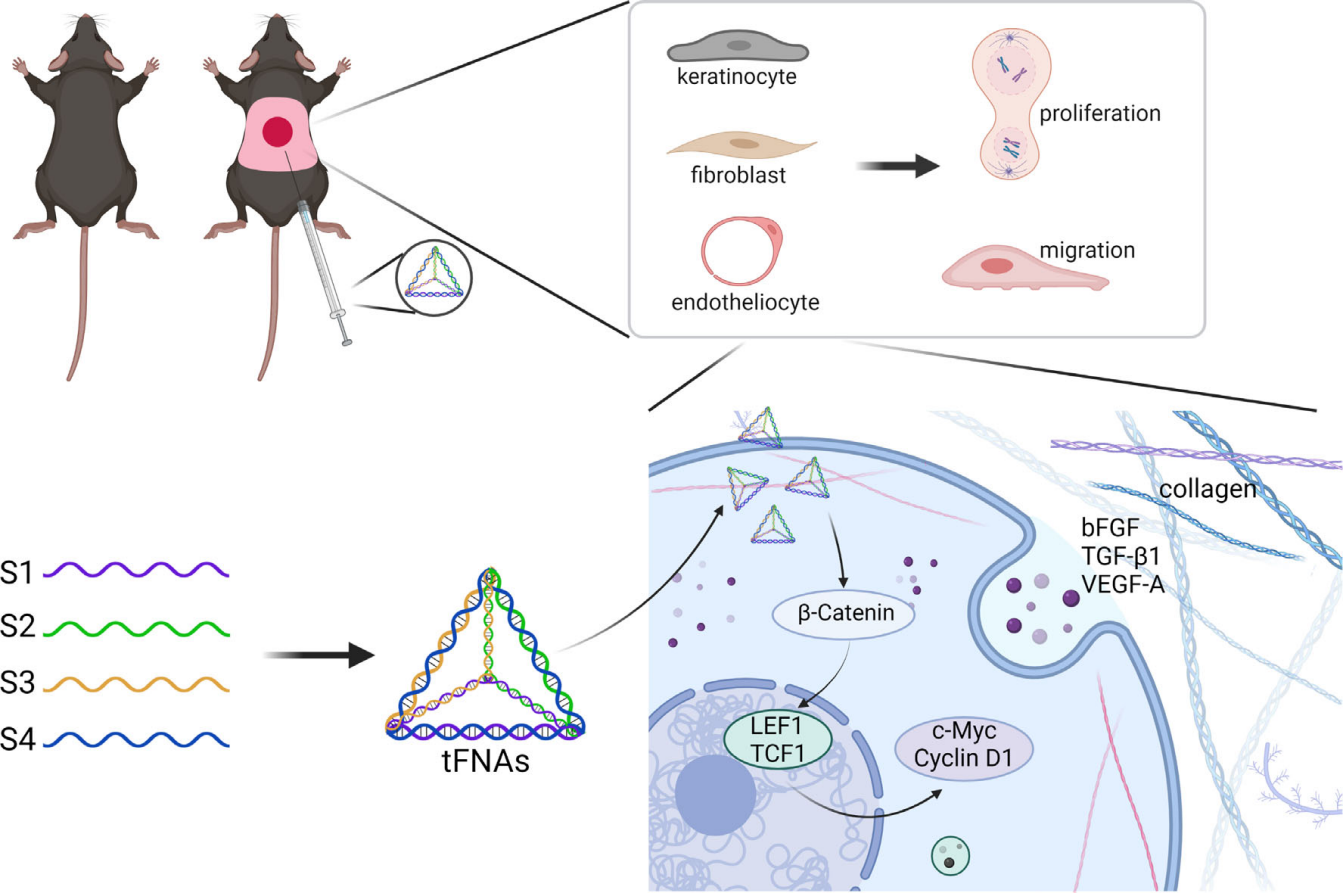
Graphical abstract of the therapeutic effects of tFNAs in diabetic wound healing (created with BioRender.com, Agreement number: AO23WPVP88)

In conclusion, our study confirmed that tFNAs can facilitate the proliferation and migration of HaCaTs, HDFs and HUVECs in a high‐glucose environment. tFNAs can accelerate diabetic wound healing by promoting epithelialization, vascularization, collagen synthesis and the secretion of growth factors via the Wnt pathway (Figure 6). These findings demonstrate that tFNAs have great potential for the treatment of diabetic wounds and pave the way for diabetic wound healing therapy.

## AUTHOR CONTRIBUTIONS

Zejing Wang and Tao Tang designed this study. Zejing Wang and Hao Lu performed all experiments. Zejing Wang and Tao Tang performed the analyses and drafted the manuscript. Lei Liu, Bohan Pan and Jiqiu Chen performed the in vivo experiments using animal models. Dasheng Cheng and Xiaoxiao Cai guided this study. Shihui Zhu initiated the study and provided funding. Shihui Zhu, Feng Zhu and Yu Sun provided full guidance throughout the research and were responsible for the study. All authors reviewed and approved the final manuscript.

## CONFLICT OF INTEREST

The authors declare no competing interests.

## Data Availability

The data supporting the findings of this study are available from the corresponding author upon reasonable request.

## References

[1] Ezhilarasu H , Vishalli D , Dheen ST , Bay BH , Srinivasan DK . Nanoparticle‐based therapeutic approach for diabetic wound healing. Nanomaterials (Basel). 2020;10(6):1234. 10.3390/nano10061234 PMC7353122 32630377

[2] Fu J , Huang J , Lin M , Xie T , You T . Quercetin promotes diabetic wound healing via switching macrophages from M1 to M2 polarization. J Surg Res. 2020;246:213‐223. 31606511 10.1016/j.jss.2019.09.011

[3] Zhang H , Nie X , Shi X , et al. Regulatory mechanisms of the Wnt/β‐catenin pathway in diabetic cutaneous ulcers. Front Pharmacol. 2018;9:1114. 30386236 10.3389/fphar.2018.01114 PMC6199358

[4] Fui LW , Lok MPW , Govindasamy V , Yong TK , Lek TK , Das AK . Understanding the multifaceted mechanisms of diabetic wound healing and therapeutic application of stem cells conditioned medium in the healing process. J Tissue Eng Regen Med. 2019;13(12):2218‐2233. 31648415 10.1002/term.2966

[5] Sanapalli BKR , Yele V , Kalidhindi RSR , Singh SK , Gulati M , Karri VVSR . Human beta defensins may be a multifactorial modulator in the management of diabetic wound. Wound Repair Regen. 2020;28(3):416‐421. 31777130 10.1111/wrr.12785

[6] Shah SA , Sohail M , Khan S , et al. Biopolymer‐based biomaterials for accelerated diabetic wound healing: a critical review. Int J Biol Macromol. 2019;139:975‐993. 31386871 10.1016/j.ijbiomac.2019.08.007

[7] Sorg H , Tilkorn DJ , Hager S , Hauser J , Mirastschijski U . Skin wound healing: an update on the current knowledge and concepts. Eur Surg Res. 2017;58(1–2):81‐94. 27974711 10.1159/000454919

[8] Yang F , Qin X , Zhang T , Lin H , Zhang C . Evaluation of small molecular polypeptides from the mantle of *Pinctada martensii* on promoting skin wound healing in mice. Molecules. 2019;24(23):4231. 10.3390/molecules24234231 PMC6930615 31766365

[9] den Dekker A , Davis FM , Kunkel SL , Gallagher KA . Targeting epigenetic mechanisms in diabetic wound healing. Transl Res. 2019;204:39‐50. 30392877 10.1016/j.trsl.2018.10.001 PMC6331222

[10] Guo S , Dipietro LA . Factors affecting wound healing. J Dent Res. 2010;89(3):219‐229. 20139336 10.1177/0022034509359125 PMC2903966

[11] Theocharidis G , Veves A . Autonomic nerve dysfunction and impaired diabetic wound healing: the role of neuropeptides. Auton Neurosci. 2020;223:102610. 31790954 10.1016/j.autneu.2019.102610 PMC6957730

[12] Saheli M , Bayat M , Ganji R , et al. Human mesenchymal stem cells‐conditioned medium improves diabetic wound healing mainly through modulating fibroblast behaviors. Arch Dermatol Res. 2020;312(5):325‐336. 31786709 10.1007/s00403-019-02016-6

[13] Mills JP , Patel P , Broekhuizen E , et al. Diabetic foot infections. Michigan Medicine University of Michigan; 2019. 31967768

[14] Chen YJ , Wu SC , Wang HC , et al. Activation of angiogenesis and wound healing in diabetic mice using NO‐delivery dinitrosyl iron complexes. Mol Pharm. 2019;16(10):4241‐4251. 31436106 10.1021/acs.molpharmaceut.9b00586

[15] Jere SW , Houreld NN , Abrahamse H . Role of the PI3K/AKT (mTOR and GSK3β) signalling pathway and photobiomodulation in diabetic wound healing. Cytokine Growth Factor Rev. 2019;50:52‐59. 30890300 10.1016/j.cytogfr.2019.03.001

[16] Han G , Ceilley R . Chronic wound healing: a review of current management and treatments. Adv Ther. 2017;34(3):599‐610. 28108895 10.1007/s12325-017-0478-y PMC5350204

[17] Zhang X , Liu N , Zhou M , Li S , Cai X . The application of tetrahedral framework nucleic acids as a drug carrier in biomedicine fields. Curr Stem Cell Res Ther. 2021;16(1):48‐56. 32321408 10.2174/1574888X15666200422103415

[18] Zhang T , Tian T , Lin Y . Functionalizing framework nucleic‐acid‐based nanostructures for biomedical application. Adv Mater. 2021;e2107820. 34787933 10.1002/adma.202107820

[19] Zhang M , Zhang X , Tian T , et al. Anti‐inflammatory activity of curcumin‐loaded tetrahedral framework nucleic acids on acute gouty arthritis. Bioact Mater. 2021;8:368‐380. 34541407 10.1016/j.bioactmat.2021.06.003 PMC8429917

[20] Xiao D , Li Y , Tian T , et al. Tetrahedral framework nucleic acids loaded with aptamer AS1411 for siRNA delivery and gene silencing in malignant melanoma. ACS Appl Mater Interfaces. 2021;13(5):6109‐6118. 33497198 10.1021/acsami.0c23005

[21] Gao S , Li Y , Xiao D , Zhou M , Cai X , Lin Y . Tetrahedral framework nucleic acids induce immune tolerance and prevent the onset of type 1 diabetes. Nano Lett. 2021;21(10):4437‐4446. 33955221 10.1021/acs.nanolett.1c01131

[22] Sirong S , Yang C , Taoran T , et al. Effects of tetrahedral framework nucleic acid/wogonin complexes on osteoarthritis. Bone Res. 2020;8:6. 32047705 10.1038/s41413-019-0077-4 PMC7010777

[23] Shao X , Cui W , Xie X , Ma W , Zhan Y , Lin Y . Treatment of Alzheimer's disease with framework nucleic acids. Cell Prolif. 2020;53(4):e12787. 32162733 10.1111/cpr.12787 PMC7162803

[24] Zhang T , Tian T , Zhou R , et al. Design, fabrication and applications of tetrahedral DNA nanostructure‐based multifunctional complexes in drug delivery and biomedical treatment. Nat Protoc. 2020;15(8):2728‐2757. 32669637 10.1038/s41596-020-0355-z

[25] Zhou M , Gao S , Zhang X , et al. The protective effect of tetrahedral framework nucleic acids on periodontium under inflammatory conditions. Bioact Mater. 2020;6(6):1676‐1688. 33313447 10.1016/j.bioactmat.2020.11.018 PMC7708773

[26] Li J , Yao Y , Wang Y , et al. Modulation of the crosstalk between Schwann cells and macrophages for nerve regeneration: a therapeutic strategy based on multifunctional tetrahedral framework nucleic acids system. Adv Mater. 2022;e2202513. 35483031 10.1002/adma.202202513

[27] Zhou M , Zhang T , Zhang B , et al. A DNA nanostructure‐based neuroprotectant against neuronal apoptosis via inhibiting toll‐like receptor 2 signaling pathway in acute ischemic stroke. ACS Nano. 2021. doi:10.1021/acsnano.1c09626 34967217

[28] Wang Y , Li Y , Gao S , Yu X , Chen Y , Lin Y . Tetrahedral framework nucleic acids can alleviate taurocholate‐induced severe acute pancreatitis and its subsequent multiorgan injury in mice. Nano Lett. 2022;22(4):1759‐1768. 35138113 10.1021/acs.nanolett.1c05003

[29] Zhao D , Liu M , Li J , et al. Angiogenic aptamer‐modified tetrahedral framework nucleic acid promotes angiogenesis in vitro and in vivo. ACS Appl Mater Interfaces. 2021;13(25):29439‐29449. 34137587 10.1021/acsami.1c08565

[30] Zhu J , Zhang M , Gao Y , et al. Tetrahedral framework nucleic acids promote scarless healing of cutaneous wounds via the AKT‐signaling pathway. Signal Transduct Target Ther. 2020;5(1):120. 32678073 10.1038/s41392-020-0173-3 PMC7366912

[31] Stunova A , Vistejnova L . Dermal fibroblasts—a heterogeneous population with regulatory function in wound healing. Cytokine Growth Factor Rev. 2018;39:137‐150. 29395658 10.1016/j.cytogfr.2018.01.003

[32] Martin P , Nunan R . Cellular and molecular mechanisms of repair in acute and chronic wound healing. Br J Dermatol. 2015;173(2):370‐378. 26175283 10.1111/bjd.13954 PMC4671308

[33] Ban E , Jeong S , Park M , et al. Accelerated wound healing in diabetic mice by miRNA‐497 and its anti‐inflammatory activity. Biomed Pharmacother. 2020;121:109613. 31707336 10.1016/j.biopha.2019.109613

[34] Yu T , Gao M , Yang P , et al. Insulin promotes macrophage phenotype transition through PI3K/Akt and PPAR‐γ signaling during diabetic wound healing. J Cell Physiol. 2019;234(4):4217‐4231. 30132863 10.1002/jcp.27185

[35] Li M , Wang T , Tian H , Wei G , Zhao L , Shi Y . Macrophage‐derived exosomes accelerate wound healing through their anti‐inflammation effects in a diabetic rat model. Artif Cells Nanomed Biotechnol. 2019;47(1):3793‐3803. 31556314 10.1080/21691401.2019.1669617

[36] Barrientos S , Stojadinovic O , Golinko MS , Brem H , Tomic‐Canic M . Growth factors and cytokines in wound healing. Wound Repair Regen. 2008;16(5):585‐601. 19128254 10.1111/j.1524-475X.2008.00410.x

[37] DiPietro LA . Angiogenesis and wound repair: when enough is enough. J Leukoc Biol. 2016;100(5):979‐984. 27406995 10.1189/jlb.4MR0316-102R PMC6608066

[38] Srifa W , Kosaric N , Amorin A , et al. Cas9‐AAV6‐engineered human mesenchymal stromal cells improved cutaneous wound healing in diabetic mice. Nat Commun. 2020;11(1):2470. 32424320 10.1038/s41467-020-16065-3 PMC7235221

[39] Wang M , Wang C , Chen M , et al. Efficient angiogenesis‐based diabetic wound healing/skin reconstruction through bioactive antibacterial adhesive ultraviolet shielding nanodressing with exosome release. ACS Nano. 2019;13(9):10279‐10293. 31483606 10.1021/acsnano.9b03656

[40] Wang C , Wang M , Xu T , et al. Engineering bioactive self‐healing antibacterial exosomes hydrogel for promoting chronic diabetic wound healing and complete skin regeneration. Theranostics. 2019;9(1):65‐76. 30662554 10.7150/thno.29766 PMC6332800

[41] Houschyar KS , Momeni A , Pyles MN , Maan ZN , Whittam AJ , Siemers F . Wnt signaling induces epithelial differentiation during cutaneous wound healing. Organogenesis. 2015;11(3):95‐104. 26309090 10.1080/15476278.2015.1086052 PMC4879891

[42] Nie X , Zhang H , Shi X , et al. Asiaticoside nitric oxide gel accelerates diabetic cutaneous ulcers healing by activating Wnt/β‐catenin signaling pathway. Int Immunopharmacol. 2020;79:106109. 31865242 10.1016/j.intimp.2019.106109

[43] Shi Y , Shu B , Yang R , et al. Wnt and Notch signaling pathway involved in wound healing by targeting c‐Myc and Hes1 separately. Stem Cell Res Ther. 2015;6(1):120. 26076648 10.1186/s13287-015-0103-4 PMC4501079

[44] Kaplani K , Koutsi S , Armenis V , et al. Wound healing related agents: ongoing research and perspectives. Adv Drug Deliv Rev. 2018;129:242‐253. 29501699 10.1016/j.addr.2018.02.007

[45] Beyene RT , Derryberry SL Jr , Barbul A . The effect of comorbidities on wound healing. Surg Clin North Am. 2020;100(4):695‐705. 32681870 10.1016/j.suc.2020.05.002

[46] Sun G , Shen YI , Harmon JW . Engineering pro‐regenerative hydrogels for scarless wound healing. Adv Healthc Mater. 2018;7(14):e1800016. 29663707 10.1002/adhm.201800016

[47] Chouhan D , Mandal BB . Silk biomaterials in wound healing and skin regeneration therapeutics: from bench to bedside. Acta Biomater. 2020;103:24‐51. 31805409 10.1016/j.actbio.2019.11.050

[48] Duangrat R , Udomprasert A , Kangsamaksin T . Tetrahedral DNA nanostructures as drug delivery and bioimaging platforms in cancer therapy. Cancer Sci. 2020;111(9):3164‐3173. 32589345 10.1111/cas.14548 PMC7469859

[49] Yang F , Li Q , Wang L , Zhang GJ , Fan C . Framework‐nucleic‐acid‐enabled biosensor development. ACS Sens. 2018;3(5):903‐919. 29722523 10.1021/acssensors.8b00257

[50] Zarei F , Soleimaninejad M . Role of growth factors and biomaterials in wound healing. Artif Cells Nanomed Biotechnol. 2018;46(sup1):906‐911. 29448839 10.1080/21691401.2018.1439836

[51] Liarte S , Bernabé‐García Á , Nicolás FJ . Role of TGF‐β in skin chronic wounds: a keratinocyte perspective. Cell. 2020;9(2):306. 10.3390/cells9020306 PMC7072438 32012802

[52] Xu Z , Han S , Gu Z , Wu J . Advances and impact of antioxidant hydrogel in chronic wound healing. Adv Healthc Mater. 2020;9(5):e1901502. 31977162 10.1002/adhm.201901502

[53] Merenda A , Fenderico N , Maurice MM . Wnt signaling in 3D: recent advances in the applications of intestinal organoids. Trends Cell Biol. 2020;30(1):60‐73. 31718893 10.1016/j.tcb.2019.10.003

[54] Huang P , Yan R , Zhang X , Wang L , Ke X , Qu Y . Activating Wnt/β‐catenin signaling pathway for disease therapy: challenges and opportunities. Pharmacol Ther. 2019;196:79‐90. 30468742 10.1016/j.pharmthera.2018.11.008

[55] Yang S , Zhang Y , Zhang Z , et al. Insulin promotes corneal nerve repair and wound healing in type 1 diabetic mice by enhancing Wnt/β‐catenin signaling. Am J Pathol. 2020;190(11):2237‐2250. 32858016 10.1016/j.ajpath.2020.08.006

